# Radical formation in individual aqueous solutions of some unsaturated fatty acids and in their mixtures

**DOI:** 10.3164/jcbn.18-8

**Published:** 2018-05-09

**Authors:** Yuji Matsui, Hideo Iwahashi

**Affiliations:** 1 Department of Chemistry, Wakayama Medical University, 580 Mikazura, Wakayama 641-0011, Japan; 2 Wakayama Physical Therapy College, 229-2 Kitano, Wakayama 649-6331, Japan

**Keywords:** polyunsaturated fatty acid-derived free radicals, ethyl radical, 7-carboxyheptyl radical, pentyl radical, 4-carboxybutyl radical

## Abstract

This study examines oxidizability in individual aqueous solutions of oleic acid, linoleic acid, α-linolenic acid, γ-linolenic acid and arachidonic acid, and in their mixtures. We used electron spin resonance (ESR), high performance liquid chromatography-electron spin resonance (HPLC-ESR) and high performance liquid chromatography-electron spin resonance-mass spectrometries (HPLC-ESR-MS). We detected 4-carboxybutyl radical derived from γ-linolenic acid, ethyl and 7-carboxyheptyl radicals derived from α-linolenic acid, and pentyl and 7-carboxyheptyl radicals derived from linoleic acid. HPLC-ESR analyses for the individual aqueous solutions of linoleic acid, α-linolenic acid, γ-linolenic acid and arachidonic acid showed less radical form for polyunsaturated fatty acids with more double bonds. On the other hand, HPLC-ESR peak height of 4-carboxybutyl radical, which form through hydrogen atom abstraction at the carbon close to the carboxy end, increased for linoleic acid/γ-linolenic acid, α-linolenic acid/γ-linolenic acid, and γ-linolenic acid/oleic acid mixtures compared to before mixing. Conversely, HPLC-ESR peak heights of ethyl, 7-carboxyheptyl and pentyl radicals, which form through hydrogen atom abstraction at the carbons close to the methyl end, decreased for linoleic acid/α-linolenic acid, linoleic acid/γ-linolenic acid, linoleic acid/oleic acid, linoleic acid/arachidonic acid, α-linolenic acid/γ-linolenic acid, and α-linolenic acid/oleic acid mixtures compared to before mixing.

## Introduction

Lipid peroxidation has received considerable attention because of its possible contribution to the damage of biological systems. When the rates of oxidation were investigated for the pure polyunsaturated fatty acids (PUFAs) or their homogeneous chlorobenzene solutions, the oxidative stability of each of these PUFAs was inversely proportional on the number of bisallylic hydrogens in the molecules.^(1–5)^ The oxidation rates of eicosapentaenoic acid and docosahexaenoic acid, two highly unsaturated fatty acids of *n*-3 series, were unexpectedly low compared to the oxidation rates of linoleic, α-linolenic, γ-1inolenic, dihomo γ-linolenic, and arachidonic acids in aqueous solution.^(6)^ Miyashita *et al.*^(3)^ also reported that polyunsaturated fatty acids are oxidatively more stable than less unsaturated fatty acids in aqueous micelles. They attributed the cause to higher flexibility of acyl chain conformation which brings about its high water permeability.^(3)^ Yazu *et al.*^(5)^ reported that the oxidation rate of eicosapentaenoate was five times slower than methyl linoleate in aqueous micelles. According to Yazu *et al.*,^(5)^ the peroxyl radical derived from eicosapentaenoate is more polar than that from methyl linoleate, and is likely to diffuse from the core to the micelle surface. This lowers the oxidizability for eicosapentaenoate in aqueous micelles by enhancing the termination reaction rate for peroxyl radicals and by reducing the rate of propagation because there may be more eicosapentaenoate peroxyl radicals at the surface than in the micelle core. Although studies on the peroxidation of polyunsaturated fatty acids have generally been performed as mentioned above, little information is available concerning the positions at which peroxidation occurs in the polyunsaturated fatty acid molecules. In order to examine the positions, we detected various lipid-derived free radicals derived from linoleic acid, α-linolenic acid or γ-linolenic acid in individual aqueous solutions of unsaturated fatty acids and their mixtures, using electron spin resonance (ESR), high performance liquid chromatography-electron spin resonance (HPLC-ESR) and high performance liquid chromatography-electron spin resonance-mass spectrometries (HPLC-ESR-MS).

## Materials and Methods

### Chemicals

Linolenic acid was from Wako Pure Chemical Industries, Ltd. (Tokyo, Japan). Linoleic acid, γ-linolenic and arachidonic acid were obtained from Sigma Aldrich Co. (St. Louis, MO). Oleic acid and α-(4-pyridyl-1-oxide)-*N*-*tert*-butylnitrone (4-POBN) were purchased from Tokyo Kasei Kogyo, Ltd. (Tokyo, Japan). Water used in these experiments was purified by passing through AUTOPURE WT101UV (Nihon Millipore Kogyo K.K., Yonezawa, Japan) after distillation. All other chemicals used were of analytical grade.

### Standard reaction mixture

In the 1.0 ml reaction mixture, there were 50 mM phosphate buffer (pH 7.4), 0.1 M 4-POBM, 0.89 mM linoleic acid (arachidonic acid, α-linolenic acid, γ-linolenic acid, oleic acid, or their mixtures), 0.38 M acetonitrile and 20 µM FeCl_3_ in a 4 ml clear glass screw thread vial with screw cap and silicone septa (diam. × thickness: 11.8 mm × 1.5 mm) (NICHIDEN RIKA-GLASS Co., Ltd. Kobe, Japan). 4-POBM is a spin-trapping agent. Reactions were performed at 30°C for 168 h. After the reaction, the reaction mixtures were applied to the ESR (or HPLC-ESR or HPLC-ESR-MS).

### Standard reaction under anaerobic conditions

 Oxygen molecules were removed by slowly bubbling nitrogen gas through the standard reaction mixture for 2 min. The reaction was then performed in sealed 10 ml glass ampoules at 30°C for 168 h.

### ESR, HPLC-ESR and HPLC-ESR-MS analyses

ESR, HPLC-ESR and HPLC-ESR-MS analyses were performed as previously described.^(7)^

## Results and Discussion

### ESR spectra of the standard reaction mixtures

**Fig. 1:**
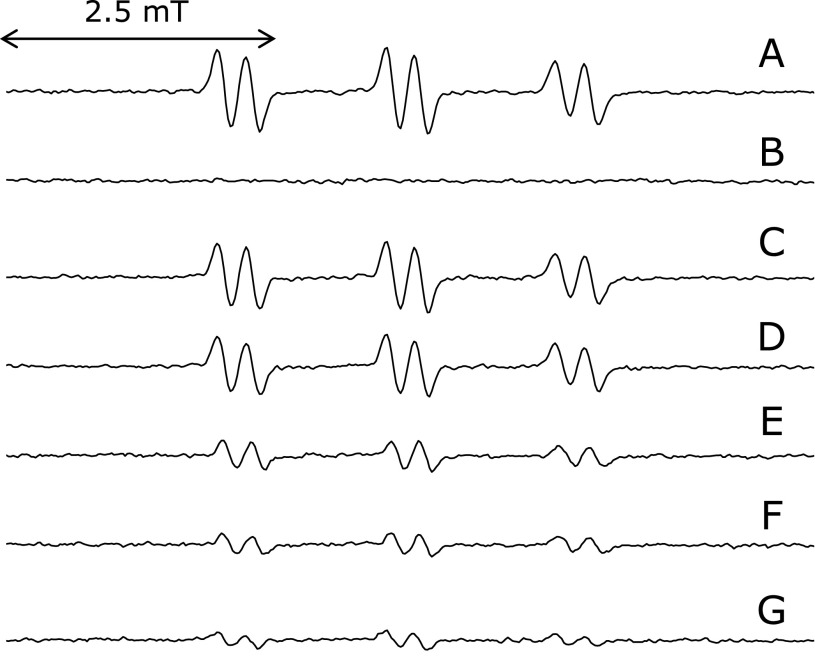
ESR spectra of the standard reaction mixtures of α-linolenic acid. Reaction and ESR conditions were as described in Materials and Methods section. In the standard reaction mixture, there were 50 mM phosphate buffer (pH 7.4), 0.1 M 4-POBN, 0.89 mM α-linolenic acid, 0.38 M acetonitrile and 20 µM FeCl_3_. (A) standard reaction mixture of α-linolenic acid. (B) without 0.89 mM α-linolenic acid. (C) without 20 µM FeCl_3_. (D) with 1 mM EDTA. (E) with 1 mM deferoxamine. (F) with 1 mM caffeic acid. (G) under anaerobic conditions.

 ESR spectrum of the standard reaction mixture (without FeCl_3,_ without α-linolenic acid, with ethylenediaminetetraacetic acid (EDTA), with deferoxamine or with caffeic acid) was measured (Fig. 1A–F) (Table 1). A prominent ESR spectrum (α^N^ = 1.58 mT and α^H^β = 0.26 mT) of 4-POBN/α-linolenic acid-derived radical adducts was observed in the standard reaction mixture (Fig. 1A). ESR peaks were hardly observed in the absence of α-linolenic acid (Fig. 1B). This indicates that the radicals formed in the standard reaction mixture are derived from α-linolenic acid. For the reaction mixture without iron, the ESR signal slightly decreased to 75 ± 16% (*n* = 9) of the standard reaction mixture (Fig. 1C). To investigate the effects of several iron chelators on the radical formation, ESR spectra were measured for the standard reaction mixture with 1 mM some iron chelators such as EDTA, deferroxamine and caffeic acid (Fig. 1D–F). The ESR peak height decreased to 65 ± 21% (*n* = 3), 33 ± 3% (*n* = 4), and 25 ± 3% (*n* = 4) of the standard reaction mixture on addition of 1 mM EDTA, deferroxamine, and caffeic acid, respectively. These results indicate that iron ions were involved in the radical formation. ESR peaks were hardly observed under the reduced O_2_ concentration (Fig. 1G). The ESR peak height decreased to 19 ± 6% (*n* = 5) of the standard reaction mixture under the reduced O_2_ concentration, indicating that oxygen molecules are involved in the radical formation.

### Time course of the ESR peak heights

**Fig. 2:**
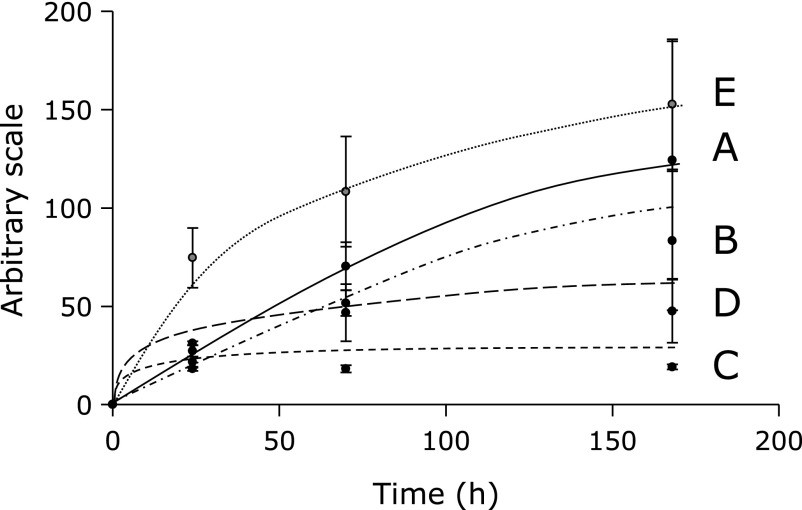
Time course of the ESR peak heights. The reaction and ESR conditions were as described in Materials and Methods section except for reaction time. In the standard reaction mixtures, there were 50 mM phosphate buffer (pH 7.4), 0.1 M 4-POBN, 0.89 mM α-linolenic acid (linoleic acid, γ-linolenic acid, α-linolenic acid and γ-linolenic acid mixture, or α-linolenic acid and linoleic acid mixture), 0.38 M acetonitrile and 20 µM FeCl_3_. Reaction times were 0, 24, 72 and 168 h. The data represent the means ± SDs of independent three measurements. (A) 0.89 mM α-linolenic acid. (B) 0.89 mM linoleic acid. (C) 0.89 mM γ-linolenic acid. (D) 0.89 mM α-linolenic acid and 0.89 mM γ-linolenic acid mixture. (E) 0.89 mM α-linolenic acid and 0.89 mM linoleic acid mixture.

Time course experiments of the ESR peak height were performed for the standard reaction mixtures of linoleic acid, α-linolenic acid and γ-linolenic (Fig. 2A–C). At 0 h, no ESR peaks were observed for linoleic acid, α-linolenic acid or γ-linolenic. The ESR peak heights of linoleic acid and α-linolenic acid gradually increased and reached plateau at 168 h. The ESR peak height of γ-linolenic acid, however, reached plateau at 24 h. Likewise, time course experiments of the ESR peak height were performed for an α-linolenic acid and γ-linolenic mixture (Fig. 2D). No ESR peaks were observed for the α-linolenic acid and γ-linolenic mixture at 0 h. The ESR peak height of the α-linolenic acid and γ-linolenic mixture gradually increased and reached plateau at 72 h. Time course experiments of the ESR peak height were also performed for a linoleic acid and α-linolenic acid mixture (Fig. 2E). The ESR peak height of the linoleic acid and α-linolenic acid mixture gradually increased and reached almost plateau at 168 h.

### HPLC-ESR analyses for the individual reaction mixtures of oleic acid, linoleic acid, α-linolenic acid, γ-linolenic acid and arachidonic acid

**Fig. 3:**
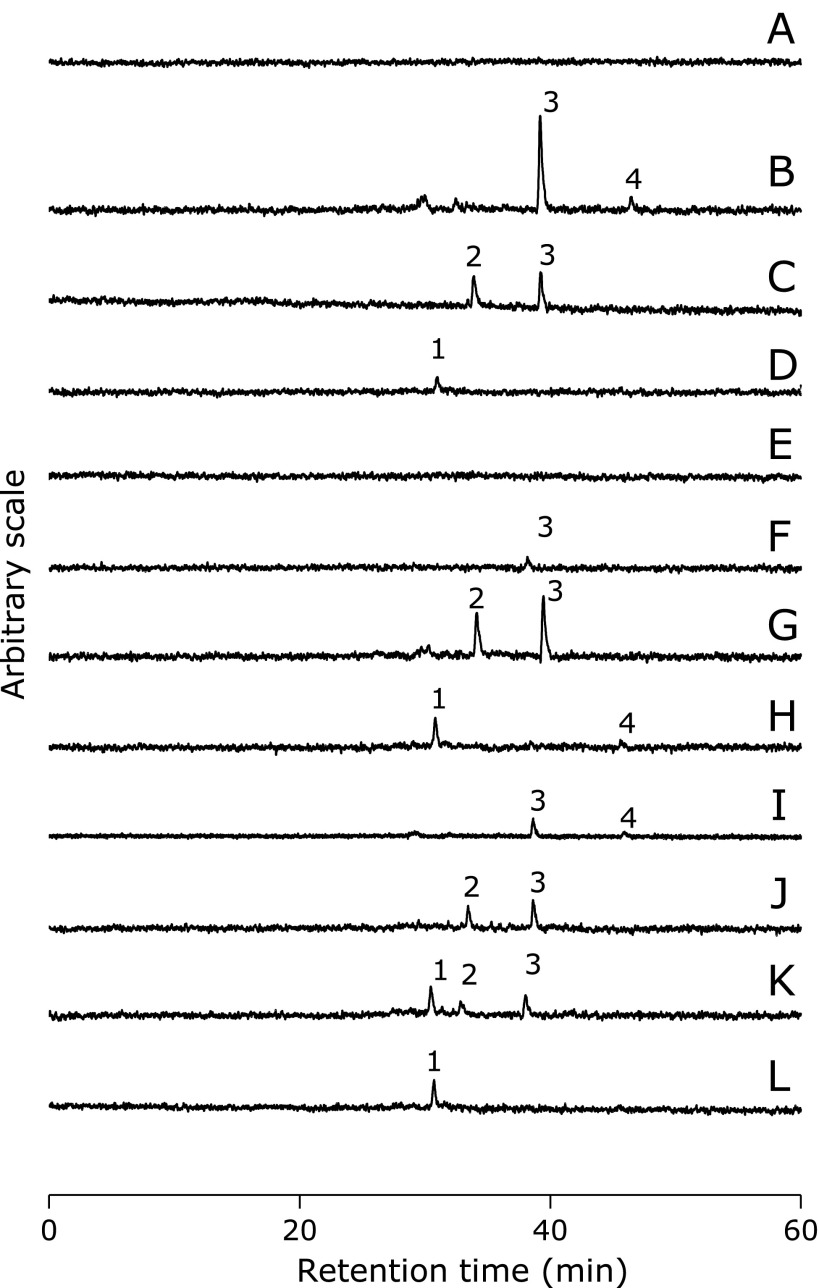
HPLC-ESR analyses of the standard reaction mixtures. Reaction and HPLC-ESR conditions were as described in Materials and Methods section. In the standard reaction mixtures, there were 50 mM phosphate buffer (pH 7.4), 0.1 M 4-POBN, 0.89 mM linoleic acid (α-linolenic acid, γ-linolenic acid, oleic acid, linoleic acid and α-linolenic acid mixture, linoleic acid and γ-linolenic acid mixture, α-linolenic acid and γ-linolenic acid mixture, linoleic acid and oleic acid mixture, α-linolenic acid and oleic acid mixture, or γ-linolenic acid and oleic acid mixture), 0.38 M acetonitrile and 20 µM FeCl_3_. (A) 0.89 mM oleic acid. (B) 0.89 mM linoleic acid. (C) 0.89 mM α-linolenic acid. (D) 0.89 mM γ-linolenic acid. (E) 0.89 mM arachidonic acid. (F) 0.89 mM linoleic acid and 0.89 mM oleic acid mixture. (G) 0.89 mM linoleic acid and 0.89 mM α-linolenic acid mixture. (H) 0.89 mM linoleic acid and 0.89 mM γ-linolenic acid mixture. (I) 0.89 mM linoleic acid and 0.89 mM arachidonic acid mixture. (J) 0.89 mM α-linolenic acid and 0.89 mM oleic acid mixture. (K) 0.89 mM α-linolenic acid and 0.89 mM γ-linolenic acid mixture. (L) 0.89 mM γ-linolenic acid and 0.89 mM oleic acid mixture.

The HPLC-ESR analyses were performed for the individual reaction mixtures of oleic acid, linoleic acid, α-linolenic acid, γ-linolenic acid and arachidonic acid. On the HPLC-ESR elution profile of the reaction mixture of linoleic acid, two prominent peaks were observed at the retention times of 38.5 ± 0.5 min (peak 3) and 45.4 ± 0.6 min (peak 4) (Fig. 3B). HPLC-ESR analyses of α-linolenic acid showed two prominent peaks at the retention times of 31.8 ± 1.5 min (peak 2) and 36.9 ± 1.7 min (peak 3) (Fig. 3C). A prominent peaks (peak 1) was observed at the retention time of 31.0 ± 0.1 min for the reaction mixture of γ-linolenic acid (Fig. 3D). HPLC-ESR peaks were hardly observed for the reaction mixture of oleic acid or arachidonic acid. (Fig. 3A and E).

### Peak areas obtained in respective HPLC-ESR analyses of the standard reaction mixtures of oleic acid, linoleic acid, α-linolenic acid, γ-linolenic acid and arachidonic acid

**Fig. 4:**
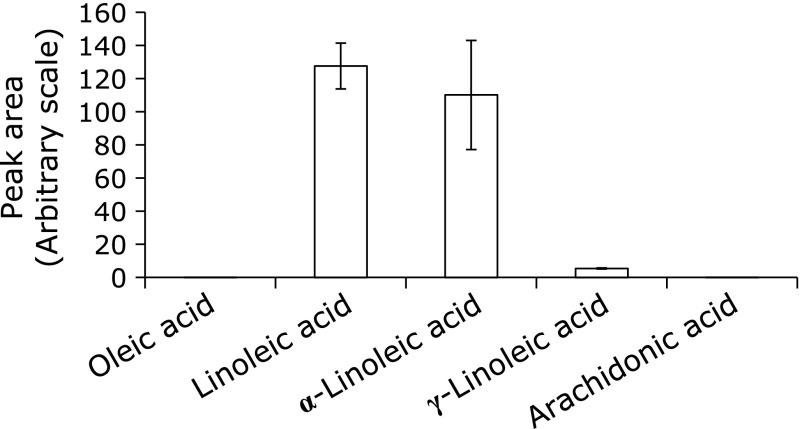
Peak area observed in respective HPLC-ESR analyses of the reaction mixtures of oleic acid, linoleic acid, α-linolenic acid, γ-linolenic acid and arachidonic acid. The peak area is sum of peaks observed for the respective fatty acid. Reaction and HPLC-ESR conditions were as described in Materials and Methods section. In the standard reaction mixtures, there were 50 mM phosphate buffer (pH 7.4), 0.1 M 4-POBN, 0.89 mM oleic acid (linoleic acid, α-linolenic acid, γ-linolenic acid, or arachidonic acid).

 Peak areas were obtained in respective HPLC-ESR analyses of the standard reaction mixtures of oleic acid, linoleic acid, α-linolenic acid, γ-linolenic acid and arachidonic acid. The peak area is the sum of peaks observed for the respective fatty acid. The peak areas (arbitrary scale) are as follows; 0 (oleic acid), 128 ± 14 (linoleic acid), 110 ± 33 (α-linolenic acid), 5.5 ± 0.4 (γ-linolenic acid), 0 (arachidonic acid) (Fig. 4). No HPLC-ESR peak was observed for oleic acid because of the absence of a methylene group connecting two or more double bonds in the molecule. On the other hand, HPLC-ESR analyses for the individual aqueous solutions of linoleic acid, α-linolenic acid, γ-linolenic acid and arachidonic acid showed that less radicals form for polyunsaturated fatty acids with more double bonds. (Fig. 4). Peak intensity of γ-linolenic acid is weak compared with α-linolenic acid, regardless of the same number in double bonds.

### HPLC-ESR-MS analyses of peaks 1, 2, 3 and 4

**Fig. 5:**
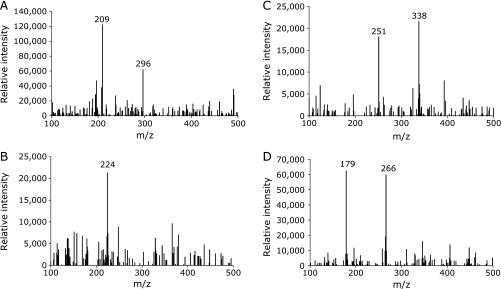
HPLC-ESR-MS analyses of peak 1, peak 2, peak 3 and peak 4. Reaction and HPLC-ESR-MS conditions were as described in Materials and Methods section. In the standard reaction mixtures, there were 50 mM phosphate buffer (pH 7.4), 0.1 M 4-POBN, 0.89 mM linoleic acid (α-linolenic acid, or γ-linolenic acid), 0.38 M acetonitrile and 20 µM FeCl_3_. (A) peak 1. (B) peak 2. (C) peak 3. (D) peak 4.

In order to find out what kinds of radicals formed in the standard reaction mixture, HPLC-ESR-MS analyses were performed for peaks 1, 2, 3 and 4 (Fig. 3). Ions at *m*/*z* 209 and *m*/*z* 296 were observed in HPLC-ESR-MS analysis of the peak 1 (Fig. 5A), suggesting that peak 1 compound was 4-POBN/4-carboxybutyl radical adduct. The ion *m*/*z* 296 corresponded to the protonated molecular ion of the 4-POBN/4-carboxybutyl radical adduct, [M + H]^+^. A fragment ion at *m*/*z* 209 corresponded to the loss of [(CH_3_)_3_C(O)N] from the protonated molecular ion. HPLC-ESR-MS analysis of peak 2 gave ions at *m*/*z* 224 (Fig. 5B), suggesting that peak 2 was 4-POBN/ethyl radical adduct. The ion *m*/*z* 224 corresponded to the protonated molecular ion of the 4-POBN/ethyl radical adduct, [M + H]^+^. HPLC-ESR-MS analysis of the peak 3 gave ions at *m*/*z* 251 and *m*/*z* 338 (Fig. 5C), suggesting that the peak 3 was 4-POBN/7-carboxyheptyl radical adduct. The ion *m*/*z* 338 corresponded to the protonated molecular ion of the 4-POBN/7-carboxyheptyl radical adduct, [M + H]^+^. A fragment ion at *m*/*z* 251 corresponds to the loss of [(CH_3_)_3_C(O)N] from the protonated molecular ion. HPLC-ESR-MS analysis of peak 4 gave ions at *m*/*z* 179 and *m*/*z* 266 (Fig. 5D), suggesting that peak 4 was the 4-POBN/pentyl radical adduct. Ion *m*/*z* 266 corresponded to the protonated molecular ion of the 4-POBN/pentyl radical adduct, [M + H]^+^. A fragment ion at *m*/*z* 179 corresponds to the loss of [(CH_3_)_3_C(O)N] from the protonated molecular ion.

It was shown that 4-carboxybutyl radical form in the control reaction mixtures of (z)-6-octadecenoic acid under irradiation at 436 nm (7.8 J cm^−2^).^(8)^ Ethyl radical reportedly forms in the photooxidation of arylcarbinols by ceric ammonium nitrate.^(9)^ Ethyl radical identification was also performed in soybean lipoxygenase-dependent peroxidation of *n*-3 polyunsaturated fatty acid.^(10)^ 7-Carboxyheptyl and pentyl radicals were detected in the reaction mixture of linoleic acid with soya bean lipoxygenase and 13-hydroperoxyoctadeca-9,11-dienoic acid with ferrous ions (or cytochrome c or haematin).^(11–13)^ 7-Carboxyheptyl radicals were also reported to form from oleic acid under flavin mononucleotide photosensitization.^(14)^

**Fig. 6:**
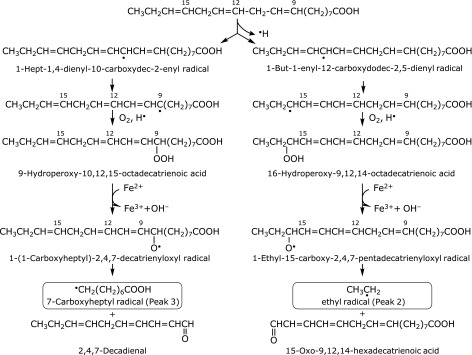
A possible reaction path for the formation of 7-carboxyheptyl radical (peak 3) and ethyl radical (peak 2) in the standard reaction mixture of α-linolenic acid.

We propose a scheme to account for the formation of the ethyl radical and 7-carboxyheptyl radical from α-linolenic acid (Fig. 6). As the ESR signal of the standard reaction mixture of α-linolenic acid without iron decreased to 75 ± 16% (*n* = 9) of the standard reaction mixture of α-linolenic acid (Fig. 1C) (Table 1), iron ions appear to catalyze the formation of 16-hydroperoxy-9,12,14-octadecatrienoic acid through the hydrogen atom abstraction at 14 carbon and 9-hydroperoxy-10,12,15-decatrienoic acid through the hydrogen atom abstraction at 11 carbon (Fig. 6). Iron complexes such as iron(IV)-oxo and iron (III)-superoxo may initiate the O_2_-activation chemistry by abstraction of an H atom from the α-linolenic acid.^(15,16)^ Furthermore, it is also suggested that iron ions were involved in the formation of ethyl radical and 7-carboxyheptyl radical, since the ESR peak height decreased to 65 ± 21% (*n* = 3) [33 ± 3% (*n* = 4) or 25 ± 3% (*n* = 4)] of the standard reaction mixture on addition of 1.0 mM EDTA (deferroxamine or caffeic acid) (Fig. 1D–F and 6) (Table 1). The ethyl radical could be a precursor of ethane, an index of lipid peroxidation.^(17–19)^

**Fig. 7:**
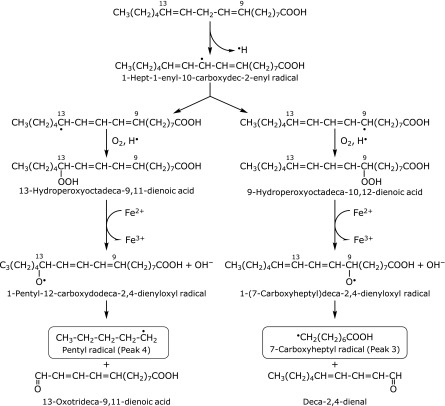
A possible reaction path for the formation of 7-carboxyheptyl radical (peak 3) and pentyl radical (peak 4) in the standard reaction mixture of linoleic acid.

**Fig. 8:**
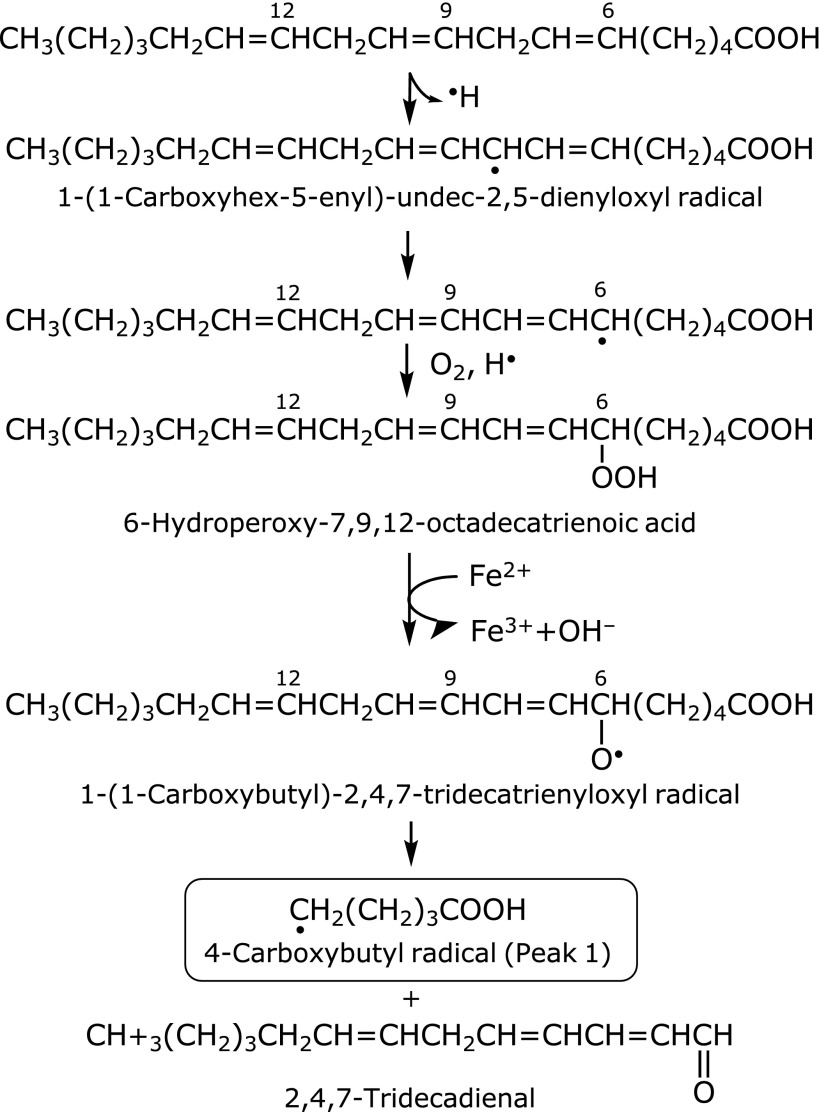
A possible reaction path for the formation of 4-carboxybutyl radical (peak 1) in the standard reaction mixture of γ-linolenic acid.

We propose schemes to account for the formation of the 7-carboxyheptyl radical and pentyl radical from linoleic acid (Fig. 7), and 4-carboxybutyl radical from γ-linolenic acid (Fig. 8). The pentyl radical could be a precursor of pentane, an index of lipid peroxidation.^(8,18,19)^

### HPLC-ESR analyses for the reactions of linoleic acid/α-linolenic acid, linoleic acid/γ-linolenic acid, linoleic acid/oleic acid, linoleic acid/arachidonic acid, α-linolenic acid/γ-linolenic acid, α-linolenic acid/oleic acid, and γ-linolenic acid/oleic acid mixtures

The HPLC-ESR analyses were performed for a linoleic acid and oleic acid mixture, a linoleic acid and α-linolenic acid mixture, a linoleic acid and γ-linolenic acid mixture, a linoleic acid and arachidonic acid mixture, an α-linolenic acid and olenic acid mixture, an α-linolenic acid and γ-linolenic acid mixture, and a γ-linolenic acid and oleic acid mixture (Fig. 3F–L). Peak height of 4-carboxybutyl radical (peak 1) increased to 248 ± 4% (linoleic acid/γ-linolenic acid) (Fig. 3H), 270 ± 80% (α-linolenic acid/γ-linolenic acid) (Fig. 3K) and 227 ± 14% (γ-linolenic acid/oleic acid) (Fig. 3L) compared to before mixing. Peak height of ethyl radical (peak 2) resulted in 119 ± 17% (linoleic acid/α-linolenic acid), 40 ± 14% (α-linolenic acid/γ-linolenic acid) and 47 ± 6% (α-linolenic acid/oleic acid) by mixing. Peak height of 7-carboxyheptyl radical (peak 3) decreased to 56 ± 8% (linoleic acid/α-linolenic acid), 6.1 ± 0.9% (linoleic acid/γ-linolenic acid), 48 ± 18% (α-linolenic acid/γ-linolenic acid), 13 ± 1% (linoleic acid/oleic acid), 58 ± 1% (α-linolenic acid/oleic acid) and 44 ± 0.2% (linoleic acid/arachidonic acid) compared to before mixing. Peak height of pentyl radical (peak 4) decreased to 0% (linoleic acid/α-linolenic acid), 36 ± 5% (linoleic acid/γ-linolenic acid), 14 ± 0.8% (linoleic acid/arachidonic acid) and 0% (linolenic acid/oleic acid) compared to before mixing.

**Fig. 9:**
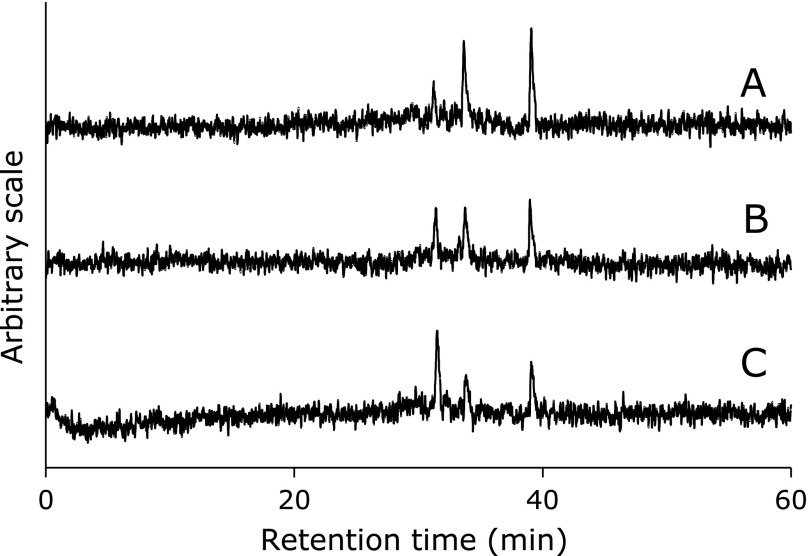
HPLC-ESR analyses of the standard reaction mixtures. Reaction and HPLC-ESR conditions were as described in Materials and Methods section except for concentration of γ-linolenic acid. In the standard reaction mixtures, there were 50 mM phosphate buffer (pH 7.4), 0.1 M 4-POBN, 0.89 mM α-linolenic acid and 0.30 mM γ-linolenic acid mixture (0.89 mM α-linolenic acid and 0.45 mM γ-linolenic acid mixture, or 0.89 mM α-linolenic acid and 0.89 mM γ-linolenic acid mixture), 0.38 M acetonitrile and 20 µM FeCl_3_. (A) 0.89 mM α-linolenic acid and 0.30 mM γ-linolenic acid mixture. (B) 0.89 mM α-linolenic acid and 0.45 mM γ-linolenic acid mixture. (C) 0.89 mM α-linolenic acid and 0.89 mM γ-linolenic acid mixture.

HPLC-ESR analyses were performed for the reaction mixture of 0.89 mM α-linolenic acid with 0.45 mM γ-linolenic acid or 0.23 mM γ-linolenic acid. Peak height of ethyl radical (peak 2) and 7-carboxyheptyl radical (peak 3) decreased with increasing γ-linolenic acid concentration (Fig. 9).

4-Carboxybutyl radical forms through the hydrogen atom abstraction at 8 carbon which is close to carboxy end in γ-linolenic acid. Ethyl radical forms through the hydrogen atom abstraction at 14 carbon in α-linolenic acid, 7-carboxyheptyl radical forms through the hydrogen atom abstraction at 11 carbon in α-linolenic acid (or linoleic acid) and pentyl radical forms through the hydrogen atom abstraction at 11 carbon in linoleic acid. Ethyl, 7-carboxyheptyl and pentyl radicals form through the hydrogen atom abstraction at the carbons which are close to methyl end. Thus, the radical formation through hydrogen atom abstraction at the carbon close to the carboxy end increased by mixing, and the radical formation through hydrogen atom abstraction at the carbons close to the methyl end decreased by mixing. Iron ions, which are close to carboxy groups of fatty acids due to electrostatic interaction, may prefer to react with bisallylic hydrogens near them, since micelles become inflexible by mixing.

## Figures and Tables

**Table 1:** Relative ESR peak heights of standard reaction mixtures of α-linolenic acid under various conditions

Conditions	ESR peak height (% standard reaction mixture)
Standard reaction mixture of α-linolenic acid	100 ± 23 (*n* = 15)
Without α-linolenic acid	0 (*n* = 3)
Without iron	75 ± 16 (*n* = 9)
With EDTA^a^	65 ± 21 (*n* = 3)
With deferroxamine^a^	33 ± 3 (*n* = 4)
With caffeic acid^a^	25 ± 3 (*n* = 4)
Anaerobic	19 ± 6 (*n* = 5)

The reaction conditions were as described in “Materials and Methods” section. ^a^1 mM EDTA (deferroxamine or caffeic acid) was added.

## Abbreviations

EDTA: ethylenediaminetetraacetic acid
ESR: electron spin resonance
HPLC-ESR: high performance liquid chromatography-electron spin resonance
HPLC-ESR-MS: high performance liquid chromatography-electron spin resonance-mass spectrometries
4-POBN: α-(4-pyridyl-1-oxide)-*N*-*tert*-butylnitrone
PUFAs: polyunsaturated fatty acids
